# Jejunum or Stomach for the Pancreatic Anastomosis After Pancreaticoduodenectomy

**DOI:** 10.1155/1997/76467

**Published:** 1997

**Authors:** Andrew L. Warshaw

**Affiliations:** Massachusetts General Hospital Harvard Medical School Wang Ambulatory Care Center-336 Boston Massachusetts 02114 USA

## Abstract

*Objective*: The authors hypothesized that pancreaticogastrostomy is safer than pancreaticojejunostomy after pancreaticoduodenectomy and less likely to be associated with a postoperative pancreatic fistula.

*Summary Background Data*: Pancreatic fistula is a leading cause of morbidity and mortality after pancreaticoduodenectomy, occurring in 10% to 20% of patients. Nonrandomized reports have suggested that pancreaticogastrostomy is less likely than pancreaticojejunostomy to be associated with postoperative complications.

*Methods*: Between May 1993 and January 1995, the findings for 145 patients were analyzed in this prospective trial at The Johns Hopkins Hospital. After giving their appropriate preoperative informed consent, patients were randomly assigned to pancreaticogastrostomy or pancreaticojejunostomy after completion of the pancreaticoduodenal resection. All pancreatic anastomoses were performed in two layers without pancreatic duct stents and with closed suction drainage. Pancreatic fistula was defined as drainage of greater than 50 mL of amylase-rich fluid on or after postoperative day 10.

*Results*: The pancreaticogastrostomy (*n*=73) and pancreaticojejunostomy (*n*=72) groups were comparable with regard to multiple parameters, including demographics, medical history, preoperative laboratory values, and intraoperative factors, such as operative time, blood transfusions, pancreatic texture, length of pancreatic remnant mobilized, and pancreatic duct diameter. The overall incidence of pancreatic fistula after pancreaticoduodenectomy was 11.7% (171145). The incidence of pancreatic fistula was similar for the pancreaticogastrostomy (12.3%) and pancreaticojejunostomy (11.1%) groups. Pancreatisc fistula was associated with a significant prolongation of postoperative hospital stay (36±5 vs. 15±1 days) (*p*<0.001). Factors significantly increasing the risk of pancreatic fistula by univariate logistic regression analysis included ampullary or duodenal disease, soft pancreatic texture, longer operative time, greater intraoperative red blood cell transfusions, and lower surgical volume (*p*<0.05). A multivariate logistic regression analysis revealed the factors most highly associated with pancreatic fistula to be lower surgical volume and ampullary or duodenal disease in the resected specimen.

*Conclusions*: Pancreatic fistula is a common complication after pancreaticoduodenectomy, with an incidence most strongly associated with surgical volume and underlying disease. These data do not support the hypothesis that pancreaticogastrostomy is safer than pancreaticojejunostomy or is associated with a lower incidence of pancreatic fistula.


### BIBLIOGRAPHY

1. Beger, H.G., Krautzberger, W. and Bittner, R. *et al.* (1985) Results of surgical treatment of necrotizing pancreatitis. *World J Surg.,* **6**, 972–979.
2. Smadja, C. and Bismuth, H.: (1986) Pancreatic debridement in acute necrotizing pancreatitis: An obsolete procedure. *Brit J Surg.,* **73**, 408–410.
3. Widdison, A.L., Alvarez, C. and Reber, H.A.: (1991) Surgical intervention in acute pancreatitis: When and how. *Pancreas.*, **6**, 544–551.
4. Uomo, G., Visconti, M. and Manes, G. *et al*: (1996) Nonsurgical treatment of acute necrotizing pancreatitis. *Pancreas.*, **12**, 142–148.
5. Bradley EL III. Debridement is rarely necessary in patients with sterile pancreatic necrosis. *Pancreas* 1996 (in press).
6. Gillaumes, S., Blanco, I., Clave, P. and Marruecos, L: (1992) Non-operative management of necrosis in acute pancreatitis. *Pancreas.*, 7, 740.
7. Andersson, R., Steinbach, R., Leveau, P. Ihse I (1994) Results of primarily non-operative treatment in severe acute pancreatitis. Abstracts of the International Hepatobiliary Pancreatic Association, p.91.
8. Uhl, W., Stupnicki, A., Schmid, S. T., Vogel, R. and Büchler, M.W. The role of surgery in severe acute pancreatitis: Lessons learned from the past. Presented at the Pancreas Club, San Diego, May 14, 1995.

Edward L Bradley, III, MD
Department of Surgery
State University of New York (Buffalo)
Buffalo General Hospital
100 High Street
Buffalo, New York 14203
United States of America

### JEJUNUM OR STOMACH FOR THE PANCREATIC ANASTOMOSIS AFTER PANCREATICODUODENECTOMY

### ABSTRACT

*Yeo, C.J., Cameron, J.L., Maher, M.M., Sauter, P.K., Zahurak, M.L., Talamini, M.A., Lillemoe, K.D. and Pitt, H.A. (1995) A prospective randomized trial of pancreaticogastrostomy versus pancreaticojejunostomy after pancreaticoduodenectomy. Annals of Surgery; 222: 580–592.*

**Objective:** **The authors hypothesized that pancreaticogastrostomy is safer than pancreaticojejunostomy after pancreaticoduodenectomy and less likely to be associated with a postoperative pancreatic fistula.**
***Summary Background Data:*** **Pancreatic fistula is a leading cause of morbidity and mortality after pancreaticoduodenectomy, occurring in 10% to 20% of patients. Nonrandomized reports have suggested that pancreaticogastrostomy is less likely than pancreaticojejunostomy to be associated with postoperative complications.**
***Methods:*** **Between May 1993 and January 1995, the findings for 145 patients were analyzed in this prospective trial at The Johns Hopkins Hospital. After giving their appropriate preoperative informed consent, patients were randomly assigned to pancreaticogastrostomy or pancreaticojejunostomy after completion of the pancreaticoduodenal resection. All pancreatic anastomoses were performed in two layers without pancreatic duct stents and with closed suction drainage. Pancreatic fistula was defined as drainage of greater than 50 mL of amylase-rich fluid on or after postoperative day 10.**
***Results:*** **The pancreaticogastrostomy ($n$=73) and pancreaticojejunostomy ($n$=72) groups were comparable with regard to multiple parameters, including demographics, medical history, preoperative laboratory values, and intraoperative factors, such as operative time, blood transfusions, pancreatic texture, length of pancreatic remnant mobilized, and pancreatic duct diameter. The overall incidence of pancreatic fistula after pancreaticoduodenectomy was 11.7% (17/145). The incidence of pancreatic fistula was similar for the pancreaticogastrostomy (12.3%) and pancreaticojejunostomy (11.1%) groups. Pancreatisc fistula was associated with a significant prolongation of postoperative hospital stay (36±5 vs. 15±1 days) ($p$<0.001). Factors significantly increasing the risk of pancreatic fistula by univariate logistic regression analysis**

**included ampullary or duodenal disease, soft pancreatic texture, longer operative time, greater intraoperative red blood cell transfusions, and lower surgical volume ($p<0.05$). A multivariate logistic regression analysis revealed the factors most highly associated with pancreatic fistula to be lower surgical volume and ampullary or duodenal disease in the resected specimen.**

***Conclusions:*** **Pancreatic fistula is a common complication after pancreaticoduodenectomy, with an incidence most strongly associated with surgical volume and underlying disease. These data do not support the hypothesis that pancreaticogastrostomy is safer than pancreaticojejunostomy or is associated with a lower incidence of pancreatic fistula.**

KEYWORDS: Pancreaticogastrostomy pancreaticojejunostomy pancreaticoduodenectomy

### PAPER DISCUSSION

The main message of this study is that it is difficult to improve on success. The authors set out to test the hypothesis that pancreaticogastrostomy is less likely to lead to an anastomotic fistula than is pancreaticojejunostomy, a finding espoused in collective experiences of proponents of using the stomach for reconstruction. They devised and planned a study with sufficient power to show a significant difference if the projected pancreatico-enteric fistula rate would be 20% and the alternate treatment reduced it to 5%. Neither projected outcome occurred; that is, the pancreaticojejunal fistula rate was only 11% and the pancreaticogastrostomy rate was 12%. The conclusion is clear that the two methods were equivalent. But equally clear is that it is hard to do better than the best, and these fistula rates are among the lowest reported.

The relatively small number of fistulas which did occur seem therefore to be less a function of the anastomotic technique than of other circumstances. The study showed in fact that, while most variables had no discernable impact on the likelihood of a pancreatic fistula occurring, there was a significant correlation with two factors. The first is with the volume of such surgery performed by the surgeon. The risk of a fistula was inversely related to the pancreaticoduodenectomy experience of the surgeon. This finding, of course, is similar – and probably causally related – to the lower mortality rates observed in high-volume vs. low-volume settings[1,2].

Second, multivariate analysis showed a greater risk of anastomotic fistula in patients with "duodenal" or "ampullary" (and bile duct) pathology as compared with a primary pancreatic disease. While the statistical analysis emphasizes this descriptor of the significant "independent variable," it is clear that the link between that type of disease and the adverse event must be – and is – the texture of the pancreas in those diseases, soft vs. firm, fibrotic or not, capable of holding sutures securely or easily disrupted. The study implies that either technique is just as good or just as bad in the high-risk circumstance. The study does not address or answer whether octreotide has a salutary role in preventing fistulas in these cases.

The authors seem to imply that because there was no difference in the outcomes from the two methods, there is no compelling reason to prefer one over the other. In fact they give no criteria for choosing. Nonetheless, there would seem to be a difference regarding the practical implications, if not the risks, between the two types of fistula. For example, I assume that the treatment of a gastric-origin fistula would require fasting and TPN until successful closure. In contrast a jejunal-origin fistula, especially from an isolated roux-en-y loop might allow the patient to eat (and go home) while the fistula continued to heal. One might therefore predict the ability of the latter technique to shorten the period of morbidity, hospitalization, and cost.

Also, while no patient in this series required a reoperation for uncontrolled sepsis and its complications, such situations do arise and usually are best controlled by amputation of the pancreatic stump and the attached/dehisced anastomosis. If the stomach is involved in the dehiscence, it is worrisome that management of the gastrostomy will be more difficult and risky.

There are still questions to be answered about the pancreatico-gastrostomy. Do these anastomoses remain patent and functional? Are there long-term complications? If there is delayed gastric emptying, as is noted in about 30% of pylorus-preserving operations[3,4], is there greater risk of pancreatico-gastric anastomotic failure? For now, we are left with not much more than our pre-existing bias to choose betweeen these two techniques. For myself, I will continue with what works for me: pancreaticojejunostomy. As Will Rogers, a sage American humorist said 60 years ago, "If it ain't broke, don't fix it".

### BIBLIOGRAPHY

1. Gordon, T.A., Burleyson, G.P., Tielsch, J.M. and Cameron, J.L. (1995) The effects of regionalization on the cost and outcome for one general high-risk surgical procedure. *Ann Surg.*, **221**, 42–49.
2. Lieberman, M.D., Kilburn, H., Lindsey, M. and Brennan, M.F. (1995) Relation of perioperative deaths to hospital volume among patients undergoing pancreatic resection for malignancy. *Ann Surg.*, **222**, 638–645.
3. Itani, K.M.F., Coleman, R.E., Akwari, O.E. and Meyers, W.C. (1986) Pylorus-preserving pancreatoduodenectomy: A clinical and physiologic appraisal. *Ann Surg.*, **204**, 655–664.
4. Yeo, C.J, Barry, M.K. and Sauter, P.K, *et al.* (1993) Erythromycin accelerates gastric emptying after pancreati coduodenectomy: A prospective, randomized, placebo-controlled trial. *Ann Surg.*, **218**, 229–238.

Andrew L Warshaw, MD
Massachusetts General Hospital
Harvard Medical School
Wang Ambulatory Care Center-336
Boston
Massachusetts 02114
United States of America

